# Hearing Loss Increases Hospitalizations among U.S. Older Adults with Heart Failure

**DOI:** 10.1093/geroni/igaf122.3785

**Published:** 2025-12-31

**Authors:** Jessica West, Hanzhang Xu, Howard Francis, Sherri Smith, Matthew Dupre

**Affiliations:** Duke University School of Medicine, Durham, North Carolina, United States; Duke University, Durham, North Carolina, United States; Duke University School of Medicine, Durham, North Carolina, United States; Duke University School of Medicine, Durham, North Carolina, United States; Duke University School of Medicine, Durham, North Carolina, United States

## Abstract

Effective communication is essential to patient-provider interactions, chronic disease self-management, and ultimately reducing excess healthcare utilization. This study investigated whether hearing loss (HL) was associated with hospitalizations among adults managing heart failure (HF). Nationally-representative prospective cohort data from the 1998-2020 Health and Retirement Study (HRS) were used to examine adults who were diagnosed with HF (n = 3,274). Hearing status was ascertained at each wave by patient-reported hearing and hearing-aid (HA) use (normal hearing, unaided HL, aided HL). Hospitalizations were assessed at each wave from participants’ reported number of hospital admissions in the prior two years. Negative binomial mixed models examined numbers of hospitalizations over time by hearing status. Among study participants (mean age 71.46 years [±10.59]), approximately 63.84% reported normal hearing, 28.53% had unaided HL, and 7.64% had aided HL. Adults with unaided HL had significantly more hospitalizations than adults with normal hearing (incidence-rate ratio [IRR]=1.14, 95% CI = 1.07-1.22, P<.001). The association was partly attenuated after adjusting for the sociodemographic and health-related characteristics of adults with HF (IRR=1.07, 95% CI = 1.00-1.14, P=.040). Adults with aided HL had no significant difference in hospitalizations compared to adults with normal hearing. Among HF patients with HL, those who were unaided had significantly more hospitalizations compared to those who wore HAs (IRR=1.26, 95% CI = 1.06-1.49, P=.008). Associations were consistent over the course of the illness and did not vary across demographic groups. Healthcare providers should consider routine hearing assessments in HF patients to identify those who may benefit from HAs to reduce their risk of potentially preventable hospitalizations.

